# A comparison of the retention of pathogenic *Escherichia coli* O157 by sprouts, leaves and fruits

**DOI:** 10.1111/1751-7915.12165

**Published:** 2014-10-29

**Authors:** Stephanie L Mathews, Rachel B Smith, Ann G Matthysse

**Affiliations:** Department of Biology, University of North CarolinaChapel Hill, NC, 27599-3280, USA

## Abstract

The retention (binding to or association with the plant) of *Escherichia coli* by cut leaves and fruits after vigorous water washing was compared with that by sprouts. Retention by fruits and leaves was similar but differed from retention by sprouts in rate, effect of wounding and requirement for poly-β,1-6-N-acetyl-D-glucosamine. *Escherichia coli* was retained by cut ends of lettuce leaves within 5 min while more than 1 h was required for retention by the intact epidermis of leaves and fruits, and more than 1 day for sprouts. Retention after 5 min at the cut leaf edge was specific for *E. coli* and was not shown by the plant-associated bacteria *Agrobacterium tumefaciens* and *Sinorhizobium meliloti.* *Escherichia coli* was retained by lettuce, spinach, alfalfa, bean, tomato, *Arabidopsis thaliana*, cucumber, and pepper leaves and fruits faster than by sprouts. Wounding of leaves and fruits but not sprouts increased bacterial retention. Mutations in the exopolysaccharide synthesis genes *yhjN* and *wcaD* reduced the numbers of bacteria retained. *PgaC* mutants were retained by cut leaves and fruits but not by sprouts. There was no significant difference in the retention of an O157 and a K12 strain by fruits or leaves. However, retention by sprouts of O157 strains was significantly greater than K12 strains. These findings suggest that there are differences in the mechanisms of *E* *coli* retention among sprouts, and leaves and fruits.

## Introduction

Infections of humans with *Escherichia coli* O157 : H7 result in bloody diarrhoea and can progress to haemolytic-uremic syndrome. The disease was first characterized in people who had acquired the bacteria by eating undercooked hamburger meat (Tuttle *et al*., 1999). In recent years, there have been several outbreaks of food-borne illness caused by *E. coli* O157 : H7 carried on plant surfaces as well as on meat products. Outbreaks due to *E. coli* O157 : H7 have been associated with alfalfa and other sprouts, lettuce and spinach leaves (Breuer *et al*., 2001; Lodato, 2002). These plants may encounter *E. coli* in the field through the use of improperly prepared manure fertilizer. They may also encounter *E. coli* through contaminated irrigation water, during harvesting through contaminated equipment or water, or in a post-harvest setting (Heaton and Jones, 2008; Berger *et al*., 2010). Once contact between the bacteria and the plant has been made, the bacteria are retained by the plant and cannot be removed by water washing (Jeter and Matthysse, 2005; Franz *et al*., 2007). Thus, these bacteria pose a risk to consumers of sprouts, leaves and fruits, which are not generally cooked prior to consumption. Several recent reviews of the interaction of *E. coli* with salad vegetables have been published (Heaton and Jones, 2008; Solomon and Sharma, 2009; Berger *et al*., 2010; Critzer and Doyle, 2010; Olaimat and Holley, 2012).

In devising techniques to reduce the contamination of salad vegetables with *E. coli* O157, it would be helpful to know whether and how the retention of the bacteria differs depending on the plant part to be consumed. In addition, knowledge of the effect of damage to the plant on bacterial retention would aid in determining conditions used to reduce contamination.

In this report, the term binding is used for attachment of bacteria to the surface of a plant tissue. The term retention is used when it is unclear if the bacteria are actually bound to the plant surface or simply trapped inside a cut tissue or natural opening. As defined here, neither retained nor bound bacteria can be removed from the plant tissue by vigorous water washing. Bacterial retention was measured at 5 min, 1 h, and 1, 3 and 4 days. Bacterial numbers after 1 day and longer reflect both initial bacterial retention and subsequent growth and/or death of bacteria retained on the plant surface.

Fett (2000) first noted in examining sprouts that bacterial biofilms on the surfaces of plants can include pathogenic bacteria. Biofilms containing *E. coli* O157 have also been described on leaves (Olmez and Temur, 2010). Significant numbers of *E. coli* O157 are known to be retained by washed alfalfa sprouts; lettuce, spinach and cabbage leaves; and cut green peppers, lettuce, carrots and cucumbers (Barak *et al*., 2002; Jeter and Matthysse, 2005; Brandl and Amundson, 2008; Heaton and Jones, 2008; Solomon and Sharma, 2009; Critzer and Doyle, 2010; Patel *et al*., 2010). Wounding has been shown to promote bacterial retention on lettuce leaves, celery and chive plants (Brandl, 2008; Harapas *et al*., 2010). In this study, bacterial retention by cut leaves (lettuce, tomato, bean and *Arabidopsis thaliana*), fruits (cherry tomato, cucumber and green bell pepper) and sprouts (alfalfa, tomato, lettuce, bean and *A. thaliana)* was examined. The results suggest that bacterial retention by sprouts differs from that by cut leaves and fruits in ways that may have an impact on methods used to reduce post-harvest contamination of vegetables.

## Results

### Specificity of bacterial interactions with cut lettuce leaves: A comparison of *E. coli*, *Agrobacterium tumefaciens* and *Sinorhizobium meliloti*

**Fig 1:**
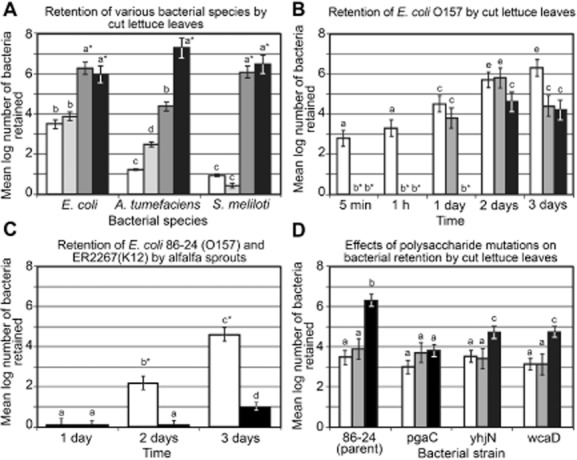
Bacterial retention by lettuce leaves and alfalfa sprouts. The mean numbers of bacteria ± standard deviation recovered from each segment are shown. In each panel bars with same letter (a, b, c, d or e) are not significantly different (*P* > 0.05). Bars with different letters and no asterisk are significantly different at *P* < 0.05. Bars with different letters and an asterisk are significantly different at *P* < 0.01. All experiments involved a minimum of three replicates and were repeated a minimum of three times on separate dates.A. Retention of various bacterial species by cut lettuce leaves after incubation for 5 min (white bars), 1 h (light grey bars), 3 days (dark grey bars) and 4 days (black bars).B. Retention of *E. coli* O157 by cut lettuce leaves. Number of bacteria retained by segments 0–1 cm (white bars), 4–5 cm (light grey bars) and 9–10 cm (dark grey bars) from the cut end. No bacteria were recovered from the 4–5 and 9–10 cm segments after 5 min or 1 h.C. Retention of *E. coli* 86-24 (O157, white bars) and ER2267 (K12, black bars) by alfalfa sprouts after incubation for 1, 2 and 3 days. Data are taken from Jeter and Matthysse (2005).D. The effect of polysaccharide mutations on bacterial retention by cut leaves incubated for 1 h (white bars), 1 day (grey bars) and 3 days (black bars).

Before making comparison of the retention of *E. coli* by sprouts, leaves and fruits, it was helpful to ascertain whether bacterial retention at cut surfaces was due to specific interactions between the bacteria and the surface or was simply non-specific trapping of small particles in the convoluted surface. In fact, many of the bacteria retained by cut leaves appear to be trapped rather than bound to the plant cells when observed with the light microscope. To examine this possibility, we compared the retention by cut lettuce leaves of *A. tumefaciens*, which generally binds to cut surfaces of dicot plants (Matthysse, 1986), and of *S. meliloti*, which binds well only to its host plant alfalfa and other members of the genus *Medicago* (Matthysse and Kijne, 1998), with the retention of *E. coli*. Both *A. tumefaciens and S. meliloti* are members of the α-*Proteobacteria*. They are approximately the same size and shape as *E. coli* and grow well on minimal media and in association with plants. In contrast to *E. coli*, both *A. tumefaciens* and *S. meliloti* showed very little retention by cut lettuce leaves in the first 5 min (Fig. 1A). For *A. tumefaciens*, the number of bacteria retained by the cut edge increased slowly to 500 after 1 h and to 2 × 10^4^ after 3 days. Both of these numbers are 10-fold less than the number of *E. coli* retained by the cut leaf. After 4 days, the numbers of *A. tumefaciens* surpassed the number of *E. coli* retained and was 10^7^ per leaf. Initially, *S. meliloti* was retained more slowly than *A. tumefaciens* or *E. coli*, but by 3 days the number of *S. meliloti* retained equalled the number of *E. coli* (10^6^ per leaf). Increased numbers of *A. tumefaciens* and *S. meliloti* at 3 and 4 days are likely due to bacterial growth on the plant surface and subsequent retention of the daughter cells. The number of bacteria retained did not increase for either *S. meliloti* or *E. coli* between 3 and 4 days. Thus, the interaction of bacteria with cut lettuce leaves is not simply a non-specific interaction involving the trapping of small particles, but depends on the species of bacterium involved.

### Characterization of the interactions of *E. coli* by cut lettuce leaves

In order to compare the interactions of *E. coli* with leaves, fruits and sprouts, we first determined the time course of bacterial retention by cut leaves. Bacterial binding and penetration to a location where they were retained during washing of cut lettuce leaves was rapid. By 5 min after placing cut lettuce leaves in a suspension of 10^5^ *E. coli* 86-24 per milliltre, more than 100 bacteria were retained by the cut edge of the leaf. Approximately 10^3^ bacteria were retained after 1 h, 10^5^ at 1 day and more than 10^6^ bacteria at 3 days (Fig. 1B). The number of bacteria retained by the first centimetre of leaf tissue from (and including) the cut edge remained constant from 3 to 7 days (data not shown). No bacteria were recovered on the leaf blade or present inside the leaf at a distance 4–5 and 9–10 cm above the cut edge after 5 min or 1 h incubation. By 1 day, about 10^4^ bacteria were recovered from the 4–5 cm segment. On and inside the leaf blade, there was an increase in the number of bacteria between 1 and 2 days to more than 10^5^ per segment; after that time, the number of bacteria on and inside the leaf remained relatively constant (Fig. 1A).

The effect of washing inoculated cut leaves each day was examined. This treatment was found to have little effect on bacterial growth and retention. At 3 days, there was no significant difference between leaves that were not washed daily (log_10_ 6.0 ± 0.2 bacteria on the cut end and log_10_ 4.1 ± 0.3 bacteria retained by the 4–5 cm segment) and washed leaves (6.0 ± 0.4 and 4.0 ± 0.5 bacteria retained respectively). This result suggests that the observed increase in bacterial numbers between days 1 and 3 was largely due to growth of bacteria already associated with leaves after 1 day incubation instead of recruitment of bacteria from the solution.

**Fig 2:**
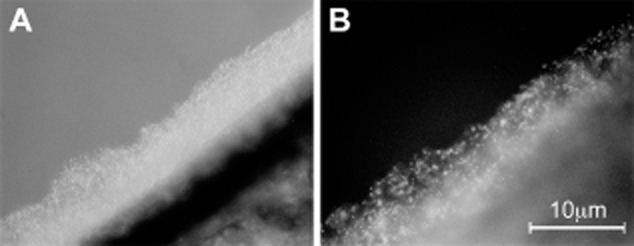
Photomicrographs of *E. coli* O157 pKT-kan on the cut edge of a lettuce leaf. (A) Light and (B) fluorescence photomicrographs. Photographed after 1 day incubation. Note that *E. coli* makes up only a fraction of the large bacterial biofilm on the edge of the leaf.

The location of the bacteria was examined by inoculating the lettuce leaf with bacteria carrying the *gfp* gene. Leaves were washed before observation to remove bacteria that were not bound to the surface or retained inside the leaf and observed in the fluorescence microscope. Most of the bacteria retained at 1 h were seen at the cut end of the central leaf vein. Few bacteria were observed on or in the intact leaf blade. After 1 day, a large number of bacteria continued to be present at the cut end of the major central vein. On the cut edge, fluorescent *E. coli* were embedded in a biofilm of other bacteria presumably derived from the bacteria originally present on the leaves (Fig. 2). Bacterial aggregates were often seen on the ends of cut veins. The majority of the *E. coli* on the cut end appeared to be bound to other bacteria as a part of a dense biofilm on the epidermis or an aggregate on the end of the vein. Some bacteria were visible on the surface of the intact blade. In lettuce, there was no evidence of any bacterial invasion of the plant tissue.

Once the time course and location of *E. coli* retention on lettuce was determined, the retention of *E. coli* by cut leaves of another dicot species that has been involved in transmission of *E. coli* O157 (spinach) was examined. Bacterial retention on cut spinach leaves obtained from plants grown in the green house and the grocery store was compared and showed no significant differences in the kinetics of bacterial retention. No significant difference in bacterial retention was seen between spinach leaves and lettuce leaves (all of the numbers of bacteria retained were within 0.5 log_10_ of those obtained with lettuce). These results suggest that bacterial retention on cut leaves does not differ significantly between similar plant species and between greenhouse and market sources.

### Bacterial retention by intact fruits

Concerns about transmission of *E. coli* O157 : H7 via salads have included the possibility that the bacteria might be transmitted by tomatoes, cucumbers and peppers. When we examined fruits incubated with *E. coli* 86-24, we found that the numbers of bacteria bound to the fruit epidermis after 3 days were small (10^2.1^ to 10^2.9^ cm^–2^) when compared with bacteria bound to epidermis of leaves (10^3.9^ to 10^4.3^ cm^–2^). After 1 day incubation, the numbers retained were less than 10 cm^–2^. However, if the fruits were washed after 1 day of incubation and then incubated in fresh water and harvested on the third day, there was no reduction in the number of bacteria retained on the epidermis at 3 days. This result suggests that the higher bacterial numbers seen on day 3 as compared with day 1 were largely due to growth of bacteria already associated with the fruits after 1 day incubation rather than recruitment of bacteria from the solution. This is similar to the observations made with cut leaves.

### Retention of *E. coli* by sprouts

Previously, Jeter and Matthysse (2005) showed that the retention of *E. coli* O157 by alfalfa sprouts grown from surface-sterilized seed was slow. Less than 10 bacteria per sprout were retained after 1 day incubation. After 2 days, the number of bacteria retained was 10^2.1^, and after 3 days it was 10^4.7^. If the sprouts were washed daily with sterile water, the number of bacteria retained at 3 days fell to 10^1.5^ (this study). Thus, unlike cut leaves or fruits, the bacteria retained by sprouts after 3 days incubation were recruited to the sprout surface from the solution after the first day of incubation. Examination of the binding of fluorescent bacteria carrying a plasmid with the *gfp* gene showed that most of the bacteria were associated with the root and that very few bacteria were retained by the shoot (Jeter and Matthysse, 2005).

### Comparison of bacterial retention by cut leaves, fruits and sprouts

Binding of *E. coli* O157 strains to alfalfa sprouts was slow and required more than 1 day for significant binding to be observed (Fig. 1C), whereas binding to cut lettuce leaves was rapid. This observation raised the question of whether the differences between bacterial binding to alfalfa sprouts and retention by cut lettuce leaves were due to differences between plant species (alfalfa and lettuce) or to differences between plant part (sprouts and cut leaves). To answer this question, retention of *E. coli* 86-24 by cut leaves and binding to sprouts of lettuce, alfalfa, bean and *A. thaliana* were measured (Table 1). In each case, the bacteria showed no significant binding to sprouts at 1 h, but did bind after 3 days. Retention by the cut end of leaves of all the species tested was significant after 5 min. These results suggest that the differences observed between bacterial retention by alfalfa sprouts and cut lettuce leaves are largely due to differences in bacterial retention by intact sprouts and cut leaves rather than to the difference between the two plant species.

**Table 1:** Binding of *E. coli* O157:H7 by spouts and cut leaves

Plant part and incubation time	Bacterial binding to (log ± mean number of bacteria retained)^1^				
	Alfalfa	Lettuce	Bean	Tomato	*A. thaliana*
Sprouts 1 h	ND^2^^a^	0.6 ± 0.6^a^	0.8 ± 0.8^a^	ND^a^	ND^a^
Sprouts 3 days	4.2 ± 0.5^b^	4.2 ± 0.5^b^	4.4 ± 0.6^b^	2.1 ± 0.9^c^	1.9 ± 0.9^c^
Cut leaves 5 min	3.3 ± 0.4^de^	3.3 ± 0.5^de^	2.7 ± 0.4^d^	3.7 ± 0.5^e^	2.6 ± 0.5^d^

1 Mean log_10_ number of bacteria retained after two washings in water per sprout or per square centimetre cut leaf segment 0–1 cm from the cut edge ± standard deviation of the mean. The average surface area of all sprouts except *A. thaliana* was between 0.5 and 2 cm^2^. *Arabidopsis thaliana* sprouts were between 0.05 and 0.2 cm^2^.

2 ND, none detected. Ten bacteria per spout could have been detected.

**a, b, c, d, e.** Numbers in the same row followed by the same superscript letters are not significantly different *P* > 0.05. Numbers in the same row followed by different superscript letters are significantly different *P* < 0.05. All experiments involved a minimum of three replicates and were repeated a minimum of three times on separate dates.

### *Escherichia coli* strain specificity of retention by sprouts, cut leaves and fruits

In earlier studies, we found that *E. coli* O157 strains bound to alfalfa sprouts; however, K12 strains of *E. coli* failed to do so (Jeter and Matthysse, 2005). To determine whether this was a general difference between the interaction of O157 and K12 strains with plant surfaces, we examined the binding of ER2267 (K12) to cut lettuce leaves. The binding of ER2267 to cut lettuce leaves was not significantly different from that of 86-24 (O157) (Table 2). Both strains bound to cut leaves in 5 min and showed similar numbers of bacteria retained after 1 h and 3 days. No significant difference in location of bacteria was observed in the fluorescence microscope. In no case was any evidence of tissue invasion by the bacteria seen. An examination of the retention of ER2267 by tomato, cucumber and pepper fruits gave similar results (Table 2). In each case, the number of bacteria retained by the intact epidermis and by the cut edge of the fruit was not significantly different between 86-24 (O157) and ER2267 (K12). Thus, the lack of retention of K12 strains appears to be limited to sprouts.

**Table 2:** Comparison of the retention of 86-24 (O157) and ER2267 (K12) strains of *E. coli* by fruits and cut lettuce leaves

Bacterial strain	Tomato fruit	Pepper fruit		Cucumber fruit		Lettuce leaves	
	Epidermis^1^	Epidermis^1^	Cut end^2^	Epidermis^1^	Cut end^2^	Epidermis^1^	Cut end^1^
86-24	2.5 ± 0.3^a^	2.5 ± 0.2^a^	3.9 ± 0.1^a^	2.5 ± 0.2^a^	3.9 ± 0.1^a^	4.3 ± 0.2^a^	6.4 ± 0.4^a^
ER2267	2.2 ± 0.3^a^	2.1 ± 0.3^a^	3.3 ± 0.7^a^	2.9 ± 1.0^a^	4.1 ± 1.0^a^	4.1 ± 0.3^a^	6.2 ± 0.2^a^

1 Mean log_10_ number of bacteria cm^–2^ retained after two washings in water.

2 Mean log_10_ number of bacteria cm^–3^.

a Numbers in the same column followed by the same superscript letters are not significantly different *P* > 0.05. All experiments involved a minimum of three replicates and were repeated a minimum of three times on separate dates.

### The effect of wounding on bacterial retention

For both leaves and fruits, wounding increased the number of bacteria retained at the cut edge of the tissue and the rate of bacterial retention. After 1 h, no bacteria could be detected on the intact epidermis of lettuce leaves while 10^3^ bacteria cm^–2^ were retained by the cut edge. After 3 days, the number of bacteria retained by the cut edge was 10^6.2^ and the number retained by the intact epidermis was 10^4.1^ (Fig. 1B). Similar to the cut edge of lettuce leaves, the cut edge of peppers and cucumbers retained larger numbers of bacteria (10^3.3^ and 10^4.1^ cm^–3^) than did intact epidermis (10^2.1^ and 10^2.9^ cm^–2^)(Table 2). In contrast to the effects of wounding on leaves and fruits, wounding of sprouts did not increase bacterial binding. No bacteria were detected bound to intact or wounded alfalfa sprouts after 1 day incubation. After 3 days, the log_10_ number of bacteria bound per sprout to intact and to wounded alfalfa sprouts was the same (5.0 ± 0.4).

### Effect of mutations in surface properties of *E. coli*

Curli and the calcium-dependent adhesin have been implicated in the retention of *E. coli* O157 by various surfaces including sprouts (Jeter and Matthysse, 2005; Torres *et al*., 2005; Boyer *et al*., 2007; Patel *et al*., 2011; Macarisin *et al*., 2012). A mutant unable to make curli (86-24A, *csgA*) and another mutant unable to make either of the calcium-dependent adhesins (AGT103A) were unaltered in retention by cut lettuce. The numbers of bacteria retained were 10^6^ and 10^4^ cm^–2^ for both the parent and mutant strains on the cut end and intact epidermis, respectively, after 3 days incubation.

Exopolysaccharides (EPSs) are known to be involved in biofilm formation by *E. coli* on some surfaces. Mutations in the synthesis of poly-β,1-6-N-acetyl-D-glucosamine (PGA) (*pgaC*), cellulose (*yhjN* also called *bcsB*) and colanic acid (*wcaD*) were previously shown to reduce the binding of *E. coli* 86-24 to alfalfa sprouts (Matthysse *et al*., 2008). We examined the effects of these mutations on the ability of *E. coli* 86-24 to be retained by cut lettuce leaves. No significant effect of any of these mutations after 5 min or 1 h incubation was observed (Fig. 1D). Unlike the parent strain, the *pgaC* mutant showed no increase in the number of bacteria retained by cut ends of lettuce leaves between 1 h and 3 days. The addition of a plasmid carrying the entire *pga* operon to the mutant bacteria partially restored binding to the cut ends of lettuce leaves (Table 3). Both the *yhjN* and *wcaD* mutants showed an increase in number of bacteria retained between 1 h and 3 days. However, after 3 days, significantly fewer mutant than parent bacteria were retained by the cut leaves for all three mutants. The addition of a plasmid carrying the *yhjN* or *wcaD* gene, respectively, to these mutants restored their ability to be retained by cut leaves. None of these mutations had any effect on the retention of bacteria by the intact leaf epidermis (Table 3).

**Table 3:** Binding and retention of bacteria by fruits and lettuce leaves after 3 days incubation

Bacterial strain	Tomato fruit	Pepper fruit		Cucumber fruit		Lettuce leaves	
	Epidermis^1^	Epidermis^1^	Cut end^1^	Epidermis^1^	Cut end^2^	Epidermis^1^	Cut end^1^
86-24	2.5 ± 0.3^a^	2.5 ± 0.2^a^	3.9 ± 0.1^a^	2.5 ± 0.2^a^	3.9 ± 0.1^a^	4.3 ± 0.2^a^	6.4 ± 0.4^a^
86-24C	2.2 ± 0.4^a^	1.4 ± 0.8^b^	4.0 ± 0.5^a^	1.4 ± 0.5^b^	4.0 ± 0.5^a^	3.9 ± 0.1^a^	3.8 ± 0.3^b^
86-24C pMM11	NM^3^	2.9 ± 0.3^a^	NM	3.0 ± 0.7^a^	NM	NM	4.9 ± 0.1^c^
86-24D	1.5 ± 0.2^b^	1.8 ± 0.5^b^	4.9 ± 0.3^a^	2.6 ± 0.2^a^	4.3 ± 0.4^a^	3.7 ± 0.3^a^	4.6 ± 0.4^b^
86-24D pBBRD	3.3 ± 0.3^c^	3.2 ± 0.5^a^	NM	NM	NM	NM	5.8 ± 0.2^a^
86-24N	1.8 ± 0.3^b^	1.0 ± 0.5^b^	4.1 ± 0.8^a^	0.8 ± 0.7^b^	4.4 ± 0.3^a^	4.1 ± 0.1^a^	4.7 ± 0.3^b^
86-24N pBBRN	3.6 ± 0.2^c^	2.2 ± 0.7^a^	NM	3.5 ± 0.6^a^	NM	NM	5.9 ± 0.4^a^

1 Mean log_10_ number of bacteria per square centimetre retained after two washings in water.

2 Mean log_10_ number of bacteria per cubic centimetre.

3 NM, not measured.

**a, b, c.** Numbers in the same column followed by the same superscript letters are not significantly different *P* > 0.05. Numbers in the same column followed by different superscript letters are significantly different *P* < 0.05. All experiments involved a minimum of three replicates and were repeated a minimum of three times on separate dates.

The effects of these three EPS mutations on bacterial binding to fruits were also examined (Table 3). Binding to the epidermis of the *yhjN* mutant was significantly reduced for all three fruits, suggesting that cellulose is involved in bacterial adhesion to the intact fruit epidermis. The roles of the other two EPSs in adhesion to the epidermis of fruits are less clear. The *wcaD* mutant was reduced in binding to tomato and pepper epidermis but showed normal binding to cucumber. The addition of a plasmid carrying the *wcaD* gene to the mutant resulted in greater retention by tomato and slightly higher retention by pepper epidermis than that seen with the parent strain. The *pgaC* mutant was reduced in binding to pepper and cucumber epidermis but showed normal binding to tomato. The addition of a plasmid carrying the entire *pga* operon to the mutant restored wild type levels of binding to pepper and cucumber epidermis (Table 3). None of the EPS mutations affected bacterial retention by the cut ends of pepper or cucumber.

## Discussion

Bacteria are retained by intact plant epidermal surfaces during water washing only if they bind to the plant surface or to other microorganisms, which are bound to the plant surface, are able to enter the plant through natural openings such as stomata or sites of emergence of lateral roots, or they possess the ability to digest the plant surface. In contrast, bacterial retention by the cut edges of leaves or fruits may not require binding or the ability to digest the plant surface. The bacteria may simply become trapped in the rough edges of the cut cells. Therefore, it was of interest to determine if the retention of *E. coli* in the cut ends of leaves depended on specific properties of the bacteria or if all bacteria showed similar retention by cut edges of plants. Retention of proteobacteria by the cut ends of lettuce leaves appears to be influenced by the bacterial species involved. The retention of *E. coli*, *A. tumefaciens* and *S. meliloti* differed from each other both in the rate of retention and the number of bacteria retained (Fig. 1A). This result suggests that the bacterial surface and/or bacterial processes such as the ability to grow on the metabolites present, the ability to withstand plant defence reactions and the ability to alter the structure of the plant surface play a role in bacterial retention and survival at the cut leaf edge.

Retention of *E. coli* O157 by cut leaves and fruits has generally been observed to be rapid occurring within hours of exposure of the leaves or fruits to the bacteria (Brandl, 2008; Castro-Rosas *et al*., 2010; 2011; Harapas *et al*., 2010; Tian *et al*., 2012). We observed that cut leaves of lettuce, spinach, alfalfa, bean and *A. thaliana* retained significant numbers of bacteria within the first 5 min of exposure. Retention of *E. coli* by alfalfa sprouts has previously been shown to be slow, requiring more than 1 day of exposure for significant numbers of bacteria to be retained (Charkowski *et al*., 2002; Jeter and Matthysse, 2005; Gomez-Aldapa *et al*., 2013b). Growth of *E. coli* O157 on mung bean sprouts has also been observed to be very slow (Gomez-Aldapa *et al*., 2013a). This appears to be a general property of sprouts as we found that retention of *E. coli* by lettuce, alfalfa, bean and *A. thaliana* sprouts was also slow.

Washing of leaves and fruits after 1 day incubation with bacteria followed by incubation in water until harvesting on the third day did not reduce the numbers of bacteria retained. This result suggests that the increase in bacterial numbers after the first day is largely due to growth of attached bacteria and that few planktonic bacteria bound to the plant surface after the first day. This was true even for intact fruit epidermis of tomato for which the numbers of bacteria retained after the first day were small (less than 100 cm^–2^). When sprouts were washed after the first and/or second day of incubation, the numbers of bacteria retained on the third day decreased approximately thousand-fold, suggesting that planktonic bacteria continued to bind to the sprout surface throughout the 3 day incubation.

Wounding has generally been observed to increase the numbers of *E. coli* retained by plant tissues (Brandl, 2008; Harapas *et al*., 2010; Castro-Rosas *et al*., 2011). We also observed that wounding increased retention of *E. coli* by leaves and fruits. However, wounding had no effect on retention of *E. coli* by sprouts.

The genome of pathogenic strains of *E*. *coli* is approximately 25% larger than that of laboratory K12 strains. Many of these additional genes are involved in human and animal infection (Blattner *et al*., 1997; Perna *et al*., 2001). A comparison of retention of O157 and K12 strains would indicate whether any of these genes are required for retention. Retention of *E. coli* 86-24 (O157) and ER2267 (K12) by cut leaves and fruits was not significantly different. However, previous studies in our laboratory have found that retention of K12 strains by sprouts was significantly less than of pathogenic strains (Jeter and Matthysse, 2005).

In order to compare mechanisms of bacterial retention by leaves, fruits and sprouts, we determined the effects in retention by leaves and fruits of mutations in genes known to be involved in retention of *E. coli* by sprouts. Although not required for bacterial binding to sprouts, both curli and the calcium-dependent adhesin are able to mediate binding of otherwise non-binding K12 strains (Torres *et al*., 2005). Mutants of 86-24 unable to produce curli or the calcium-dependent adhesins and a double mutant unable to produce either of these were unaltered in retention by cut lettuce. The presence of curli has been observed to increase bacterial retention by leaves in some cases (Patel *et al*., 2011; Fink *et al*., 2012; Macarisin *et al*., 2012). In other cases, curli have apparently activated plant defence responses and were associated with a decrease in bacterial survival on leaves (Seo and Matthews, 2012).

In contrast to curli and the calcium-dependent adhesins, the absence of certain EPSs appeared to reduce bacterial retention by many of the plant parts examined here. As previously shown, the EPSs colonic acid, cellulose and PGA are all required for optimal binding of *E. coli* O157 to alfalfa sprouts (Matthysse *et al*., 2008). This is a direct binding of *E. coli* to the plant surface or to previously bound *E. coli*. No other bacteria were present on the plant surface to which the *E. coli* could bind. Mutants lacking cellulose or colonic acid retained some ability to bind to sprouts, but mutants lacking PGA were unable to bind at a detectable level. Barak and colleagues (2007) mutated homogenous genes in *Salmonella enterica* to determine the effect of retention on sprouts and found that mutants lacking colonic acid showed no change in binding to sprouts. However, binding to sprouts was reduced in mutants lacking cellulose.

Retention of these same mutants by the cut ends of leaves was unaffected after short incubation times but reduced after 3 days, suggesting that the absence of these EPSs reduced growth (or increased death) of bacteria retained during the first day. The mutants were unaltered in retention by intact lettuce leaf surfaces. Cellulose-minus mutants have also been observed to be unaltered in retention by spinach leaves (Macarisin *et al*., 2012). Bacterial retention on tomato epidermis was decreased for *yhjN* (cellulose-minus) and *wcaD* (colonic acid-minus) mutants. Retention on pepper epidermis was decreased for all EPS mutants examined (*pgaC*, *yhjN* and *wcaD*). Retention on cucumber epidermis was decreased for *pgaC* and *yhjN* mutants. Bacterial retention by cut surfaces of pepper and cucumber was not affected in these mutants. Unlike sprouts, PGA was not required for retention of detectable numbers of bacterial on leaves or fruits. The results in this study suggest that PGA is required for bacterial retention by sprouts and that EPSs are involved in bacterial retention and growth (or prevention of death) on lettuce leaf cut ends, and tomato, pepper and cucumber epidermis.

Bacterial retention by sprouts appears to differ from that by leaves and fruits in the rate, effect of wounding, requirement for pathogenic strains and requirement for PGA. The reasons for these differences are unknown. Possible explanations include limited availability of nutrients and absence of other bacteria on sprouts, differences in the composition of the plant surfaces and the difference between growing (sprouts) as compared with mature tissues (leaves and fruits). The results suggest that reducing post-harvest contamination of sprouts and leaves and fruits may require different techniques. Early and frequent washing is more likely to be effective on sprouts than on cut leaves or fruits. Inhibiting PGA synthesis or blocking the site to which it binds is also more likely to be effective on sprouts.

## Experimental procedures

### Bacterial strains and media

Strains and plasmids used in this study are listed in Table 4. *Escherichia coli* were grown in Luria–Bertani (LB) broth or on LB or MacConkey agar at 37°C. *Agrobacterium tumefaciens* and *S. meliloti* were grown in LB at 23°C. Antibiotics were added to media at the following concentrations: kanamycin 20 μg ml^–1^, chloramphenicol 30 μg ml^–1^, carbenicillin 50 μg ml^–1^, streptomycin 100 μg ml^–1^ and rifampicin 50 μg ml^–1^. The plasmids pBC*gfp* (Matthysse *et al*., 1996) and pKT-Kan (Barak *et al*., 2002) that carry *gfp* were introduced into the bacteria by calcium-mediated transformation to produce fluorescent bacteria. Other plasmids were also introduced in the same way. For the purpose of complementing the mutations, *yhjN* and *wcaD* were each cloned into the broad-host range vector pBBR1-mcs05 (Kovach *et al*., 1995). The *yhjN* gene was amplified by polymerase chain reaction (PCR) using the following primers: 5′-CGACTG*AAGCTT*GGATCCATGAAAAGAAAACTATTCTGGATTTGTC-3′ and 5′-CGTAG*CGGCCG*CATGGGGCCCTTACTCGTTATCCGGGTTAAGACGACG-3′, and cloned in frame between the HinDIII and EagI sites of the vector (the restriction sites in the primers are indicated by italics). The *wcaD* gene was amplified by PCR using the following primers: 5′-GC*GGTACC*TACAGTGGACAACAGATGC-3′ and 5′-CG*GAGCTC*GCTAAGCAACATGTTCTTATTG-3′, and cloned in frame between the KpnI and SacI sites of the vector.

**Table 4:** Properties of bacterial strains and plasmids used in this research

Bacterial strain or plasmid	Relevant properties	Source or reference
86-24	*E. coli* O157:H7 Sm^r^ Nal^r^	Torres and colleagues (2002)
8624A	86-24 *csgA*, Sm^r^ Ap^r^	Jeter and Matthysse (2005)
8624N	*yhjN* (cellulose^–^) Carb^r^	Matthysse and colleagues (2008)
8624D	*wcaD* (colonic acid^-^) Carb^r^	Matthysse and colleagues (2008)
8624C	*pgaC* (PGA^–^) Carb^r^	Matthysse and colleagues (2008)
AGT103A	86-24 *cah::cat, cah::tet csgA*, Sm^r^ Cm^r^ Tc^r^ Ap^r^	Torres and colleagues (2002)
ER2267	K12 F′ *proA^+^B^+^ lacI^q^ Δ(lacZ)M15 zzf::mini-Tn*10 (Kan^R^)/*Δ(argF-lacZ)U169 glnV44* e14^-^(McrA^-^) *rfbD1? recA1 relA1? endA1 spoT1? thi-1 Δ(mcrC-mrr)114::IS10*, Km^r^	New England Biolabs
*Agrobacterium tumefaciens* C58R	wild-type, spontaneous Rif^r^ mutant	Matthysse lab collection
*Sinorhizobium meliloti* 1021R	wild-type (cellulose^-^), spontaneous Rif^r^ mutant	Matthysse lab collection
pBC*gfp*	Cm^r^, *gfp*-expressing plasmid	Matthysse and colleagues (1996)
pKT-kan	Km^r^, *gfp*-expressing plasmid	Barak and colleagues (2002)
pMM11	pBBR1mcs Cm^r^ contains the *bps* operon of *Bordetella bronchiseptica* cloned behind the *lac* promoter	Parise and colleagues (2007)
pBBR1mcs-05	Broad-host range cloning vector	Kovach and colleagues (1995)
pBBRD	pBBR1mcs-05 carrying *wcaD* cloned behind the *lac* promoter Gent^r^	This study
pBBRN	pBBR1mcs-05 carrying *yhjN* cloned behind the *lac* promoter Gent^r^	This study

Ap, ampicillin; Carb, carbenicillin; Cm, chloramphenicol; Gent, gentamycin; Km, kanamycin; Nal, nalidixic acid; Rif, rifampicin; Sm, streptomycin; Tet, tetracylcine.

### Bacterial retention by leaves

Romaine lettuce (*Lactuca sativa* cv. green towers) was grown in the green house. Leaves 15–20 cm long were cut from the middle of the plant. Cut leaves were washed in water, and bacterial retention was measured by placing them in groups of three leaves in 10 ml of a suspension of approximately 10^5^ bacteria ml^−1^ in a sealed plastic bag for the indicated interval (5 min, 1 h, 1 day, 2 days or 3 days) at 25°C. The bag was inverted to ensure that all the surfaces of the leaves were wet and then placed with only the bottom 2–3 mm of the cut end of the leaf in the bacterial suspension. Leaves remained green during the 3 day incubation. At the time of harvest, leaves were washed in 700 ml of water, the desired segment excised, washed again in 5 ml of water in a vial that was inverted six to eight times and ground in 1 ml of water using a mortar and pestle. The cut end was the first centimetre from the cut at the basal end of the leaf including the cut site. Measurements of bacterial retention by intact epidermis were made using 1 cm segments from 5–6 and 10–11 cm from the cut basal edge of the leaf. Homogenates were plated on MacConkey agar containing appropriate antibiotics to determine viable cell counts. Homogenization was not harmful to the bacteria as similar numbers of viable bacteria were recovered from suspensions of bacteria plated directly and after homogenization with leaves (the bacteria were added directly to the leaf segments in the mortar). Colonies of *E. coli* O157 grown on MacConkey agar were characterized by their red colour. Homogenates of plant samples that were not inoculated with *E. coli* failed to produce any red colonies under these conditions. Leaves that were washed during incubation were removed from the bacterial suspension and washed two times in 700 ml of water and the placed in fresh water for continued incubation.

For inoculation of cut leaves of other species, tomato (*Lycopersicum esculentum*) cv. Rutgers, bean (*Phaseolus vulgaris*) cv. Kentucky wonder and *A. thaliana* ecotype Columbia plants were grown in the green house. Spinach (*Spinacia oleracea*) was grown in the green house or purchased from a local supermarket. Leaves were inoculated and processed as described for cut lettuce leaves.

Microscopic studies of bacteria on plant surfaces were carried out as previously described (Jeter and Matthysse, 2005; Torres *et al*., 2005). Both the abaxial and adaxial sides of the leaf were examined.

### Bacterial retention by sprouts

Measurements of bacterial binding to alfalfa sprouts were carried out as previously described (Jeter and Matthysse, 2005). Alfalfa sprouts (*Medicago sativa*) were obtained by germinating surface sterilized seeds. After 1 day of germination, four sprouts (between 0.3 and 0.8 cm long) were placed in plastic dishes with 5 ml of sterile deionized water. Bacteria were diluted and added to the germinated seeds to a final concentration of approximately 5 × 10^3^ bacteria ml^–1^ and the dishes incubated at 25°C for the indicated time. In order to wash sprouts during incubation, the liquid containing the free bacteria was poured out of the dish and replaced with fresh water, the spouts agitated gently, the liquid removed and fresh water added. This washing procedure was repeated once. At the time of harvest, sprouts were placed in vials containing 5 ml of sterile deionized water and washed by inversion; this was repeated with a fresh vial and 5 ml of water. The sprouts were then homogenized in 1–5 ml of washing buffer or water and plated on MacConkey agar containing antibiotics as appropriate to determine viable cell counts.

Sprouts of tomato, lettuce, bean and *A. thaliana* were obtained from seeds and processed as described for alfalfa, except that *A. thaliana* seeds were stored at 4°C for 3 days before germinating in 2 ml of water. *Arabidopsis thaliana* seeds were germinated until the root began to protrude from the seed coat and were then inoculated with bacteria.

### Bacterial retention by fruits

Cherry tomatoes (*L. esculentum*), green bell peppers (*Capsicum annuum*) and unwaxed cucumbers (*Cucumis sativus*) were obtained at a local supermarket. They were washed with water and placed in a suspension of approximately 10^5^ bacteria ml^–1^ at 25°C for the indicated interval (5 min, 1 day or 3 days). For washing of fruit during incubation, the fruit was removed from the bacterial suspension and washed two times in 700 ml of water and then placed in fresh water for continued incubation.

To measure bacterial retention by cut cucumbers or peppers, fruit was cut immediately before immersing it in the bacterial suspension. After 1–3 days of incubation at 25°C, the fruits were washed in 700 ml of water and 1 cm^2^ piece of epidermis or 1 cm^2^ surface area by 0.1 cm deep pieces of tissue from the cut end were removed. The tissue pieces were then washed in 5 ml of water. They were ground in 1 ml of water using a mortar and pestle. Homogenates were plated on MacConkey agar containing appropriate antibiotics to determine viable cell counts. Homogenates of plant samples that were not inoculated with *E. coli* failed to produce any red colonies on MacConkey agar.

## References

[b1] Barak JD, Whitehand LC, Charkowski AO (2002). Differences in attachment of *Salmonella enterica* serovars and *Escherichia coli* O157:H7 to alfalfa sprouts. Appl Environ Microbiol.

[b2] Barak JD, Jahn CE, Gibson DL, Charkowski AO (2007). The role of cellulose and O-antigen capsule in the colonization of plants by *Salmonella enterica*. Mol Plant Microbe Interact.

[b3] Berger CN, Sodha SV, Shaw RK, Griffin PM, Pink D, Hand P, Frankel G (2010). Fresh fruit and vegetables as vehicles for the transmission of human pathogens. Environ Microbiol.

[b4] Blattner FR, Plunkett G, Bloch CA, Perna NT, Burland V, Riley M (1997). The complete genome sequence of *Escherichia coli* K-12. Science.

[b5] Boyer RR, Sumner SS, Williams RC, Pierson MD, Popham DL, Kniel KE (2007). Influence of curli expression by *Escherichia coli* 0157:H7 on the cell's overall hydrophobicity, charge, and ability to attach to lettuce. J Food Prot.

[b6] Brandl MT (2008). Plant lesions promote the rapid multiplication of *Escherichia coli* O157:H7 on postharvest lettuce. Appl Environ Microbiol.

[b7] Brandl MT, Amundson R (2008). Leaf age as a risk factor in contamination of lettuce with *Escherichia coli* O157:H7 and *Salmonella enterica*. Appl Environ Microbiol.

[b8] Breuer T, Benkel DH, Shapiro RL, Hall WN, Winnett MM, Linn MJ (2001). A multistate outbreak of *Escherichia coli* O157:H7 infections linked to alfalfa sprouts grown from contaminated seeds. Emerg Infect Dis.

[b9] Castro-Rosas J, Santos Lopez EM, Gomez-Aldapa CA, Gonzalez Ramirez CA, Villagomez-Ibarra JR, Gordillo-Martinez AJ (2010). Incidence and behavior of *Salmonella* and *Escherichia coli* on whole and sliced zucchini squash (*Cucurbitapepo*) fruit. J Food Prot.

[b10] Castro-Rosas J, Gomez-Aldapa CA, Acevedo-Sandoval OA, Gonzalez Ramirez CA, Villagomez-Ibarra JR, Chavarria HN (2011). Frequency and behavior of *Salmonella* and *Escherichia coli* on whole and sliced jalapeno and serrano peppers. J Food Prot.

[b11] Charkowski AO, Barak JD, Sarreal CZ, Mandrell RE (2002). Differences in growth of *Salmonella enterica* and *Escherichia coli* O157:H7 on alfalfa sprouts. Appl Environ Microbiol.

[b12] Critzer FJ, Doyle MP (2010). Microbial ecology of foodborne pathogens associated with produce. Curr Opin Biotechnol.

[b13] Fett WF (2000). Naturally occurring biofilms on alfalfa and other types of sprouts. J Food Prot.

[b14] Fink RC, Black EP, Hou Z, Sugawara M, Sadowsky MJ, Diez-Gonzalez F (2012). Transcriptional responses of *Escherichia coli* K-12 and O157:H7 associated with lettuce leaves. Appl Environ Microbiol.

[b15] Franz E, Visser AA, Van Diepeningen AD, Klerks MM, Termorshuizen AJ, van Bruggen AH (2007). Quantification of contamination of lettuce by GFP-expressing *Escherichia coli* O157:H7 and *Salmonella enterica* serovar Typhimurium. Food Microbiol.

[b16] Gomez-Aldapa CA, Rangel-Vargas E, Bautista-De LH, Vazquez-Barrios ME, Gordillo-Martinez AJ, Castro-Rosas J (2013a). Behavior of enteroaggregative Escherichia coli, non-O157-shiga toxin-producing *E. coli*, enteroinvasive *E. coli*, enteropathogenic *E. coli* and enterotoxigenic *E. coli* strains on mung bean seeds and sprout. Int J Food Microbiol.

[b17] Gomez-Aldapa CA, Rangel-Vargas E, Torres-Vitela MR, Villarruel-Lopez A, Castro-Rosas J (2013b). Behavior of non-O157 Shiga toxin-producing *Escherichia coli*, enteroinvasive *E. coli*, enteropathogenic *E. coli*, and enterotoxigenic *E. coli* strains on alfalfa sprouts. J Food Prot.

[b18] Harapas D, Premier R, Tomkins B, Franz P, Ajlouni S (2010). Persistence of *Escherichia coli* on injured vegetable plants. Int J Food Microbiol.

[b19] Heaton JC, Jones K (2008). Microbial contamination of fruit and vegetables and the behaviour of enteropathogens in the phyllosphere: a review. J Appl Microbiol.

[b20] Jeter C, Matthysse AG (2005). Characterization of the binding of diarrheagenic strains of *E. coli* to plant surfaces and the role of curli in the interaction of the bacteria with alfalfa sprouts. Mol Plant Microbe Interact.

[b21] Kovach ME, Elzer PH, Hill DS, Robertson GT, Farris MA, Roop RM, Peterson KM (1995). Four new derivatives of the broad-host-range cloning vector pBBR1MCS, carrying different antibiotic-resistance cassettes. Gene.

[b22] Lodato RJ (2002). Sprout-associated outbreaks. Ann Intern Med.

[b23] Macarisin D, Patel J, Bauchan G, Giron JA, Sharma VK (2012). Role of curli and cellulose expression in adherence of *Escherichia coli* O157:H7 to spinach leaves. Foodborne Pathog Dis.

[b24] Matthysse AG (1986). Initial interactions of *Agrobacterium tumefaciens* with plant host cells. Crit Rev Microbiol.

[b25] Matthysse AG, Kijne JW, Spaink HP, Kondorosi A, Hooykaas P (1998). Attachment of *Rhizobiaceae* to plant cells. The Rhizobiaceae.

[b26] Matthysse AG, Stretton S, Dandie C, McClure NC, Goodman AE (1996). Construction of GFP vectors for use in gram-negative bacteria other than *Escherichia coli*. FEMS Microbiol Lett.

[b27] Matthysse AG, Deora R, Mishra M, Torres AG (2008). Polysaccharides cellulose, poly-beta-1,6-n-acetyl-D-glucosamine, and colanic acid are required for optimal binding of *Escherichia coli* O157:H7 strains to alfalfa sprouts and K-12 strains to plastic but not for binding to epithelial cells. Appl Environ Microbiol.

[b28] Olaimat AN, Holley RA (2012). Factors influencing the microbial safety of fresh produce: a review. Food Microbiol.

[b29] Olmez H, Temur S (2010). Effects of different sanitizing treatments on biofilms and attachment of *Escherichia coli* and *Listeria monocytogenes* on green leaf lettuce. Food Sci Technol.

[b30] Parise G, Mishra M, Itoh Y, Romeo T, Deora R (2007). Role of a putative polysaccharide locus in *Bordetella* biofilm development. J Bacteriol.

[b31] Patel J, Millner P, Nou X, Sharma M (2010). Persistence of enterohaemorrhagic and nonpathogenic *E. coli* on spinach leaves and in rhizosphere soil. J Appl Microbiol.

[b32] Patel J, Sharma M, Ravishakar S (2011). Effect of curli expression and hydrophobicity of *Escherichia coli* O157:H7 on attachment to fresh produce surfaces. J Appl Microbiol.

[b33] Perna NT, Plunkett G, Burland V, Mau B, Glasner JD, Rose DJ (2001). Genome sequence of enterohaemorrhagic *Escherichia coli* O157:H7. Nature.

[b34] Seo S, Matthews KR (2012). Influence of the plant defense response to *Escherichia coli* O157:H7 cell surface structures on survival of that enteric pathogen on plant surfaces. Appl Environ Microbiol.

[b35] Solomon EB, Sharma M, Sapers GM, Solomon EB, Matthews KR (2009). Microbial attachment and limitations of decontamination methodologies. The Produce Contamination Problem: Causes and Solutions.

[b36] Tian J-Q, Bae Y-M, Choi N-Y, Kang D-H, Heu SLSY (2012). Survival and growth of foodborne pathogens in minimally processed vegetables at 4 and 15°C. J Food Sci.

[b37] Torres AG, Perna NT, Burland V, Ruknudin A, Blattner FR, Kaper JB (2002). Characterization of Cah, a calcium-binding and heat-extractable autotransporter protein of enterohaemorrhagic *Escherichia coli*. Mol Microbiol.

[b38] Torres AG, Jeter C, Langley W, Matthysse AG (2005). Differential binding of *Escherichia coli* O157:H7 to alfalfa, human epithelial cells, and plastic is mediated by a variety of surface structures. Appl Environ Microbiol.

[b39] Tuttle J, Gomez T, Doyle MP, Wells JG, Zhao T, Tauxe RV, Griffin PM (1999). Lessons from a large outbreak of *Escherichia coli* O157:H7 infections: insights into the infectious dose and method of widespread contamination of hamburger patties. Epidemiol Infect.

